# Responsiveness Expressions of Bitter Taste Receptors Against Denatonium Benzoate and Genistein in the Heart, Spleen, Lung, Kidney, and Bursa Fabricius of Chinese Fast Yellow Chicken

**DOI:** 10.3390/ani9080532

**Published:** 2019-08-06

**Authors:** Enayatullah Hamdard, Zengpeng Lv, Jingle Jiang, Quanwei Wei, Zhicheng Shi, Rahmani Mohammad Malyar, Debing Yu, Fangxiong Shi

**Affiliations:** College of Animal Science and Technology, Nanjing Agricultural University, Nanjing 210095, China

**Keywords:** denatonium benzoate, genistein, chicken, ggTas2Rs, bitter taste receptors

## Abstract

**Simple Summary:**

In chickens, bitter taste is the most significant biological taste disrupter; it is believed to protect chickens against consuming poisonous/toxic materials and considered a warning signal prior to ingestion. The bitter taste receptors’ extraoral expression information is deficient in chicken, and denatonium benzoate is extensively used as a bitter taste receptor agonist in different cells. Our results found that qRT-PCR showed a high level of dose-dependent expressions of ggTas2Rs in the starter and grower stages in the heart, spleen, lungs, and kidneys, while the dose-dependent expressions were lower in the bursa Fabricius. The growth performance of the selected organs significantly (and unexpectedly) improved upon the administration of denatonium benzoate 5 mg/kg and genistein 25 mg/kg treatments, while the gains in organ weights were impaired in the groups given denatonium benzoate 20 mg/kg and 100 mg/kg, respectively.

**Abstract:**

The present study was conducted to investigate the responsiveness expressions of ggTas2Rs against denatonium benzoate (DB) and genistein (GEN) in several organs of the Chinese Fast Yellow Chicken. A total of 300 one-day-old chicks that weighed an average of 32 g were randomly allocated into five groups with five replicates for 56 consecutive days. The dietary treatments consisted of basal diet, denatonium benzoate (5 mg/kg, 20 mg/kg, and 100 mg/kg), and genistein 25 mg/kg. The results of qRT-PCR indicated significantly (*p* < 0.05) high-level expressions in the heart, spleen, and lungs in the starter and grower stages except for in bursa Fabricius. The responsiveness expressions of ggTas2Rs against DB 100 mg/kg and GEN 25 mg/kg were highly dose-dependent in the heart, spleen, lungs, and kidneys in the starter and grower stages, but dose-independent in the bursa Fabricius in the finisher stage. The ggTas2Rs were highly expressed in lungs and the spleen, but lower in the bursa Fabricius among the organs. However, the organ growth performance significantly (*p* < 0.05) increased in the groups administered DB 5 mg/kg and GEN 25 mg/kg; meanwhile, the DB 20 mg/kg and DB 100 mg/kg treatments significantly reduced the growth of all the organs, respectively. These findings indicate that responsiveness expressions are dose-dependent, and bitterness sensitivity consequently decreases in aged chickens. Therefore, these findings may improve the production of new feedstuffs for chickens according to their growing stages.

## 1. Introduction

In chickens, bitter taste is one of the most significant senses for choosing and consuming feeds, alongside their olfactory and visual senses [1,2,3]. Taste signals have been associated to food recognition and avoidance, as well as feed or liquid intake in different species of animals [4,5,6,7]. Bitter taste provokes an aversive reaction and is assumed to protect chickens against consuming poisons and harmful toxic substances. The age effects in humans were found to be almost exclusively generic and taste sensitivity was found to decline with age, although the level of bitterness differs depending on taste quality and is never compound-specific within a taste [8]. Chickens demonstrate bitter taste sensitivity despite having only three bitter taste receptors: ggTas2R1, ggTas2R2, and ggTas2R7. It has been shown that chickens have a well-developed sense of taste and only three of the aforementioned bitter receptors have been investigated [9]. In chickens, an insufficient number of studies have been performed to investigate growth-related taste loss and its subsequent effect on the animal’s production. Additionally, behavioral experiments were conducted and found that day-old and immature chicks were more susceptible against salt and sour taste qualities than the adults [10]. It is meaningful to elucidate the bitter taste sensitivity of chicken because of the different nutrient requirements and prerequisites during their growing stages. Bitter molecules detected by the ggTas2R family of G-protein-coupled receptors (GPCRs) were involved in perceiving potentially toxic compounds [11,12,13]. The commercial feed factories produce several categories of chicken feed according to their growth stages such as starter, grower, and finisher. When the taste buds are counted, birds have few compared to humans and other mammals. For example, humans have approximately 9000 taste buds, chickens have between 250–350 taste buds, and pigeons have only 37–75 [14,15,16]. Furthermore, in chickens, the gustatory and extra-gustatory mechanisms of involving taste signaling have been shown recently [1,17,18,19].

Denatonium benzoate (DB) is intensely bitter and non-toxic, which can be detected by human taste receptors [20]. Denatonium benzoate has been demonstrated widely as a bitter taste agonist and used to activate bitter taste receptors on many cell types, including tastes cells [21]. A previous study indicated that denatonium increased cholecystokinin release through Ca^2+^ influx in enteroendocrine STC-1 cells [22]. After oral glucose administration in diabetic mice, a prior gavage of denatonium attenuated blood glucose levels through the increased secretion of glucagon-like peptide-1 [23]. In addition, exposure to denatonium quickly suppressed the ongoing intake and delayed gastric emptying in rodents [24,25]. Apparently, genistein is mainly derived from soy products, which contain a phytochemical with isoflavone structure that is found in an extensive variety of foods, legumes, animal forages, and particularly in soybeans. Genistein (GEN) has protective effects against atherosclerosis, cardiovascular risk, and type 2 diabetes, which are attributed to its antioxidant activity; furthermore, dietary GEN can enhance the growth performance of livestock [26,27,28,29].

In recent years, the majority of reports on the expression of extra-gustatory taste receptors obviously suggested that their role is not restricted to taste perception in the mouth and gastrointestinal tract. Taste receptors have additionally been recognized in the respiratory system [30,31] and gastrointestinal tracts of mammals, in the male reproductive system, and in the brain, as well as in the heart [32,33]. Currently, the direct involvement of the human bitter taste receptor TAS2R38 in the detection of minimum bacterial sensing molecules was suggested [34]. One of the most recent additions of scientific investigations is the expression of bitter taste receptors in human and animal hearts [32,35]. Remarkably, the whole heart cDNA of neonatal rats analyzed by qRT-PCR, the seven bitter taste receptors genes, and two genes encoding the umami receptor subunits, Tas1R1 and Tas1R3, were identified as expressed in hearts. However, many research studies on the influence of bitter taste receptors materials on cardiac tissue observe need to be warranted [36].

Taste sensitivity in chickens is lower compared to that of mammals. It has been reported that chickens respond to several tastants, and these responses are conserved from post-hatching until adulthood [10,37,38,39,40,41]. Nevertheless, the relationship between taste sensitivity and number of taste buds in various chicken breeds remain unclear. It has been clarified recently that Tas2Rs receptors are also expressed in the extraoral tissues of the chicken [35,42]. The molecular mechanism of bitter molecules by their receptors is slightly complicated and less studied. However, bitter taste receptors (Tas2Rs or T2Rs) are developing as novel regulators of native immunity in the respiratory tract [43].

Recent studies findings indicate that T2Rs are extensively expressed in several parts of the human body, and have been identified to be involved in respiratory system physiology, the gastrointestinal tract, and the endocrine system, and T2Rs may play regulatory roles in the mentioned areas of the body [35,41,44,45]. In contrast, GPCRs facilitate the sensations of bitter, sweet, and umami tastes in mammals and chickens [46]. Moreover, the gene expression compilation collection (http://www.ncbi.nlm.nih.gov/geo) also confirmed that T2Rs are widely expressed in other human tissues such as the heart, brain, skeletal muscle, endometrium, liver, omental adipose tissue, nasal cavity, lung, and different cell types (chemosensory cells, smooth muscle cells, endothelial cells, epithelial cells, and inflammatory cells) [47].

The detection of taste thresholds and their identification is crucial for studying the potential effects of Tas2Rs on chicken feeding behavior. Interestingly, the chicken’s genome contains only three bitter taste receptors, which are responsible for bitterness identification, as described previously [48]. The presence of a minimum lower number of bitter taste receptors makes the chicken a significant minimalistic model for an understanding of vertebrate taste necessities [49]. However, there is limited knowledge about the expressions of bitter taste receptors, and no study has yet investigated the bitter taste responsiveness expressions in the extra-gustatory organs of chickens. Therefore, the objectives of this research were to investigate the bitter taste receptors’ (ggTas2Rs) responsiveness expressions against different doses of denatonium benzoate, genistein and compare the ggTas2Rs mRNA expressions levels among different organs in the starter, grower, and finisher stages of Chinese Fast Yellow Chicken.

## 2. Materials and Methods

### 2.1. Chemicals

Denatonium benzoate 98% was purchased from Adamas Reagent Co Ltd. (Nanjing, China), Genistein 98% was purchased from Kai Meng. Co Ltd. (Xi’an, China) and stored at room temperature. RNAose (phenol 38%), trichloroethane, isopropyl alcohol, alcohol absolute, and DNA/RNA-ase free water were purchased collectively for RNA extraction from TAKARA BIO INC (Nojihigashi 7-4-38, Kusatsu, Shiga Japan), while PrimeScript™ RT reagent Kit with gDNA Eraser (Perfect Real Time, Cat. # RR047A v201710Da) and TB Green™ Premix Ex Taq™ (Tli RNase H Plus, Cat. # RR420A v201710Da) were both purchased from TaKaRa (Dalian, China).

### 2.2. Birds and Procedures

The experimental protocol and procedures were designed and approved in accordance with the Guidelines for the Care and Use of Animals prepared by the Institutional Animal Care and Ethical Committee for Nanjing Agricultural University, Nanjing, China (Permit Number: SYXK (Su) 2019–0036). A total of three hundred (300), 1-day-old Chinese Fast Yellow chicks at the average weight of 32 ± 5 g were randomly allocated into five (5) groups with five replicates of 12 chicks in each. In order to find the dose-dependent comparison expressions of bitter taste receptor genes against DB, we designed the experiment with different doses of denatonium benzoate from low levels to high levels; the experiment groups were as follows: the control, denatonium benzoate 5 mg/kg (Low Dose), denatonium benzoate 20 mg/kg (Medium Dose), denatonium benzoate 100 mg/kg (High Dose), and genistein 25 mg/kg (GEN 25 mg/kg). All were reared under the ventilated chicken house in which the light remained 16-h light: 8-h dark, humidity was approximately 40–45% and formulated feed (Table 1) was offered ad libitum with freely available tap water for 56 consecutive days.

### 2.3. Feed Formulation and Mixing Procedure

We purchased the basal diet from ADM factory (Nanjing, China) with respective ingredients (Table 1); then, we mixed the basal diet feed with the different treatments through an electric feed mixing machine available in the Nanjing Agricultural University animal house. According to the experimental design, five (5) types of treatments were prepared: the basal diet (Control), denatonium benzoate 5 mg/kg (Low Dose), denatonium benzoate 20 mg/kg (Medium Dose), denatonium benzoate 100 mg/kg (High Dose), and genistein 25 mg/kg. The chemicals and organic materials were mixed appropriately with the help of an electric mixer and provided ad libitum for the feeding of chicken until the end of the experiment, respectively.

### 2.4. Organs Weight Measurements

A total of 10 chickens in each group (two chickens/replicate) were used in each stage of killing to collect and measure the heart, spleen, lung, kidney, and bursa Fabricius weights at Day 7 (starter stage), Day 28 (grower stage), and Day 56 (finisher stage), respectively (Table 3).

### 2.5. Sample Collection and RNA Extraction

On days 7, 28, and 56 (the starter, grower, and finisher stages) tissues from the heart, spleen, lung, kidney, and bursa Fabricius were collected and kept in an −80 °C freezer until RNA extraction. Afterward, the total RNA for RT-PCR and real-time PCR was extracted and purified from frozen collected tissues using RNAose (TAKARA BIO INC, Nojihigashi 7-4-38, Kusatsu, Shiga Japan), which includes gDNA Eraser (Perfect Real Time) for the elimination of genomic (g) DNA according to the manufacturer’s protocols.

### 2.6. Primer Design and RT-PCR

Initially, first-strand cDNA was synthesized by reverse transcription (RT) with the application of 2.0 ug of total RNA with or without reverse transcriptase using the PrimeScript™ RT reagent Kit with gDNA Eraser (Perfect Real Time, Cat. # RR047A v201710Da) in accordance with the manufacturer’s instructions. Gene-specific primers for ggTas2R1 (Accession no. AB249766.1), ggTas2R2 (Accession no. AB249767.1), ggTas2R7 (Accession no. NM_001080719.1) and the housekeeping gene (β-actin) (Accession no. NM_205518.1) were generated with the aid of the nucleotide database of The National Center for Biotechnology Information (NCBI) [50], according to their published cDNA sequences (Table 2). The target genes and the housekeeping gene were synthesized by the Sangon company and applied for real-time PCR (Table 2). Amplicon lengths for real-time PCR were between 102–162 bp. The PCR mixture consisted of 2 µL of a cDNA template diluted in a ratio of 1:3, 10 µL of TB Green premix Ex Taq (Tli RNase H Plus) (2×), 0.4 µL of forward primer (10 µM), 0.4 µL of reverse primer (10 µM), 0.4 µL of ROX Reference Dye 1 or Dye 2 (50×) and 6.8 µL of DNA/RNA enzyme-free water in a final volume of 20 µL (TaKaRa, Dalian, China). Entire PCR reactions were performed in 96-well reaction plates on a 7500 Real-time PCR instrument (Applied Biosystems, ABI, USA), and all the genes were repeated six times under the following conditions: ha old stage (95 °C for 30 s), a PCR stage (40 cycles of 95 °C/2 min, 60 °C/34 s) apparently to verify the amplification of a single product, while a stage with a temperature increment (melt curve stage) was conducted to generate a melting curve under the following conditions: (95 °C/15 s, 60 °C/1 min), followed by a temperature increment of 95 °C/15 s.

### 2.7. Housekeeping Gene for Internal Control (β-actin)

We investigated the Ref-Finder online database (http://www.leonxie.com/rferencegene.php) to choose the most constant housekeeping gene as an internal control for the real-time PCR analysis. The mentioned database consists of various computational programs (geNorm, Norm-finder, Best-Keeper, and the comparative ΔΔCt method). We calculated the relative gene expression (arbitrary units) utilizing the 2^-ΔΔCt^ method and normalized the relative abundance to tested candidate reference genes. Promising housekeeping genes were statistically tested for significant differences among various tested tissues, developmental time points, and their interaction using JMP Pro 10 software (SAS Institute, 2006, Cary, NC) [49,51]. The geometric average of β-actin was found to be the most stable and significant reference gene with no significant differences (*p* > 0.05) among the target organs (heart, spleen, lung, kidney, and bursa Fabricius) on days 7, 28, and 56 using one-way ANOVA for analysis.

### 2.8. Determination of mRNA Expression by Real-Time PCR Using the Comparative ΔΔCt Method

To confirm and validate the target gene expressions for the first time, the data were subjected to new ∆∆Ct fold-change calculations [52], and statistical analysis were carried out to compare the expression of the ggTas2Rs target genes in heart, spleen, lung, kidney, and bursa Fabricius in the starter, grower, and finisher stages of the chicken. Finally, the efficiencies of all the tested genes and the reference gene were calculated. Cycle threshold (Ct) values for every sample were calculated using the ΔCt (Δ cycle threshold) procedure [53]. Gene expression was normalized against the geometric average of β-actin. Changes in mRNA determination were analyzed by comparing the relative expression among the genes in the heart, spleen, lung, kidney, and bursa Fabricius in different denatonium benzoate treatments, GEN, and a control group. Each stage’s relative expression data were analyzed separately, and consequently, each target gene in a single selected organ was compared across all of the growing stages, respectively. Primers and gene accession numbers are described in Table 3, as described in detail in previous publications [49,54].

### 2.9. Statistical Analysis

#### 2.9.1. Organ Weight Measurements 

Weight measurements for five (5) selected organs were described previously [51]. Significant differences between treatment groups and the control group were analyzed by one-way analysis of variance (ANOVA) followed by Duncan’s post hoc test. A *p* < 0.05 was considered as statistically significant, and subsequently marked with (a, b, c, d). Value = MEAN ± SEM, weight unit (g).

#### 2.9.2. Gene Expression

For relative gene expression analysis of the genes (ggTas2R1, ggTas2R2, and ggTas2R7), for each organ compared the chosen control gene (β-actin) in all the tissues (heart, spleen, lung, kidney, and bursa Fabricius) in different growing stages using one-way ANOVA. In addition, multiple comparison among means of ggTas2R1, ggTas2R2, ggTas2R7, and the β-actin gene in each group were calculated using Dunnett’s test (marked with a, b, c, d, and e), and *p* < 0.05 was considered significant, as shown in the figures, respectively. An alpha level of 0.05 was set for all the tests. These statistical analyses were conducted with GraphPad Prism 6 and IBM SPSS Statistics, version 20 software (SPSS Inc., Chicago, IL, USA).

## 3. Results

### 3.1. Organ Weight Measurements

The results on the heart weight gained showed that the GEN and DB 5 mg/kg (Low Dose) groups significantly (*p* < 0.05) gained more weight compared to other DB and control groups (Table 3). Conversely, the live body weight and organ weights gained for the heart in the denatonium benzoate 100 mg/kg (High Dose) significantly (*p* < 0.05) decreased in the starter and grower stages, but not in the finisher stage (Table 3). However, no significant differences were observed among other treatments (Table 3). Meanwhile, the live body weight and spleen weight gained in the GEN group in all the growth stages significantly (*p* < 0.05) increased compared to the medium dose of DB 20 mg/kg. However, the spleen weight in the denatonium benzoate 20 mg/kg (Medium Dose) group increased in the grower and finisher stages compared to the DB High-Dose group, respectively (Table 3). However, the spleen weight of the control group increased in the grower stage among all the groups (Table 3). Lung weight gaining also significantly (*p* < 0.05) increased in the GEN and DB Medium-Dose groups in the starter, grower, and finisher stages, while it significantly decreased in the DB High-Dose group in the starter stage and surprisingly increased in the DB Low-Dose group compared to the DB High-Dose group at the grower and finisher stages (Table 3). Furthermore, the kidney weight in the GEN group in all the growing stages increased significantly (*p* < 0.05) and decreased in the DB 100 mg/kg (High-Dose) group in the grower and finisher stages among the groups, while it increased in the DB 5 mg/kg (Low-Dose group) in the starter and grower stages compared to the other DB doses, respectively (Table 3). Finally, the weight gained for bursa Fabricius remarkably increased in the DB 5 mg/kg (Low-Dose group) in the grower and finisher stages, while it decreased in the GEN and DB 20 mg/kg (Medium-Dose) groups in all three stages of growing, respectively (Table 3). The interaction between feed and stages was declared in data analysis; there was lower interaction in the starter and grower stages, while higher interaction was observed in the finisher stage. Meanwhile, the live body weight and organ weights subsequently increased, while taste sensitivity decreased, respectively (Table 3).

### 3.2. Detection of ggTas2Rs Responsiveness Expressions Against Denatonium Benzoate and Genistein

#### 3.2.1. mRNA Responsiveness Expressions of ggTas2Rs in Chicken Heart

**Figure 1:**
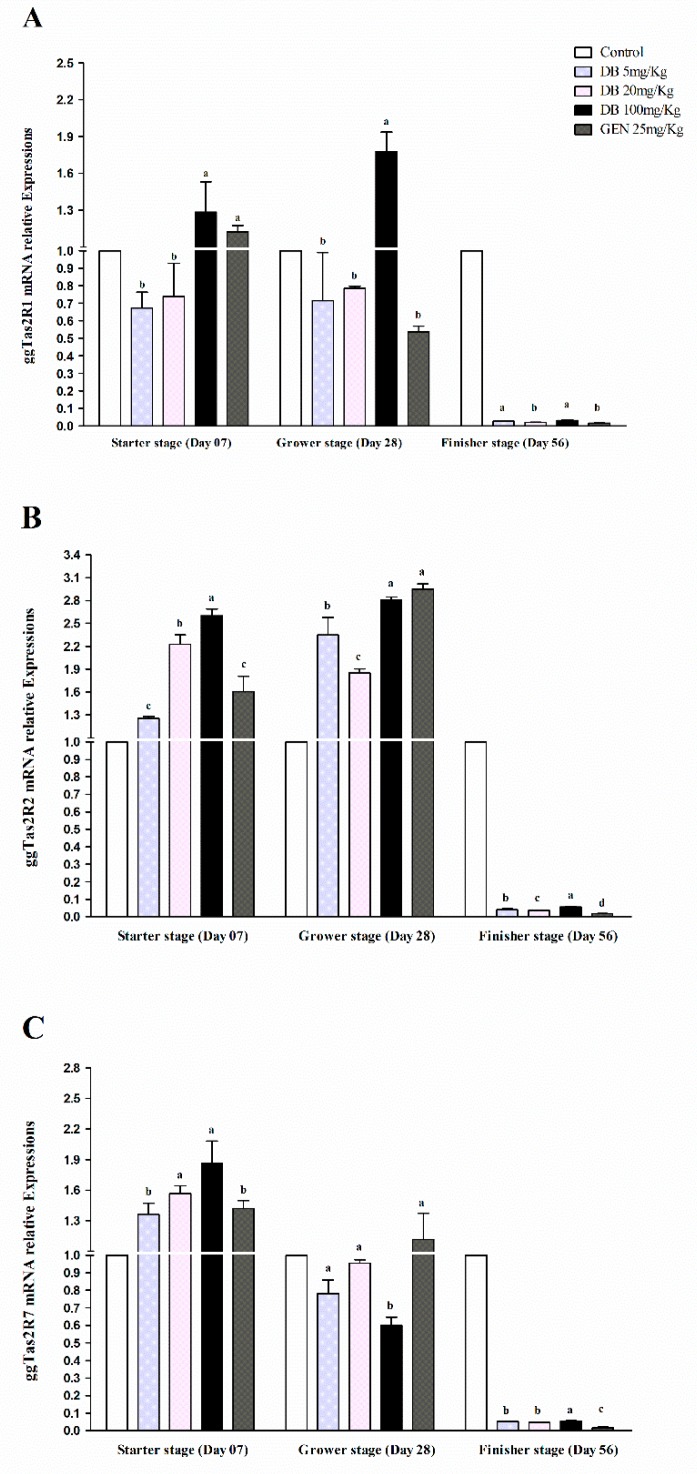
Real-time PCR analysis of (**A**) ggTas2R1, (**B**) ggTas2R2, and (**C**) ggTas2R7 of bitter taste receptors showing their relative mRNA expressions against different doses of DB and GEN in the hearts of chickens in different stages of growth (Day 7, Day 28, and Day 56). The relative mRNA abundance of ggTas2Rs in different growth stages with the heart serving as the control (relative expression set to 1; n = 6). Values are presented as the mean of relative expressions ± SEM. Differences between groups within a gene means those without a common letter differ significantly (*p* < 0.05); differences between the tested tissue (heart of chicken) and the control tissue (heart) within a gene means that those with marks (a, b, c) differ significantly (*p* < 0.05) from the control group by ANOVA.

Real-time PCR analysis showed that the expressions of ggTas2R1, ggTas2R2, and ggTas2R7 in chicken hearts was significantly (*p* < 0.05) higher in the starter and grower stages in all the treated doses of denatonium benzoate and genistein compared to the finisher stage (Figure 1A–C). While comparing different growing stages, the ggTas2R1, ggTas2R2, and ggTas2R7 genes were dose-dependent and highly expressed in GEN and DB 100 mg/kg (High Dose) in the starter and grower stages among other treatments and consequently similarly lower expressed in the finisher stage (Figure 1A–C). However, the expressions of ggTas2R1, ggTas2R2, and ggTas2R7 in the DB 5 mg/kg (Low Dose), DB 20 mg/kg (Medium Dose), and GEN groups were almost similar in the starter and grower stages, except for ggTas2R2 in the GEN group in the grower stage (Figure 1B and Figure 1A–C). The expressions of ggTas2Rs in all the groups were lower in the finisher stage, and no significant differences were observed among them.

#### 3.2.2. mRNA Responsiveness Expression of ggTas2Rs in the Spleen of Chicken 

**Figure 2:**
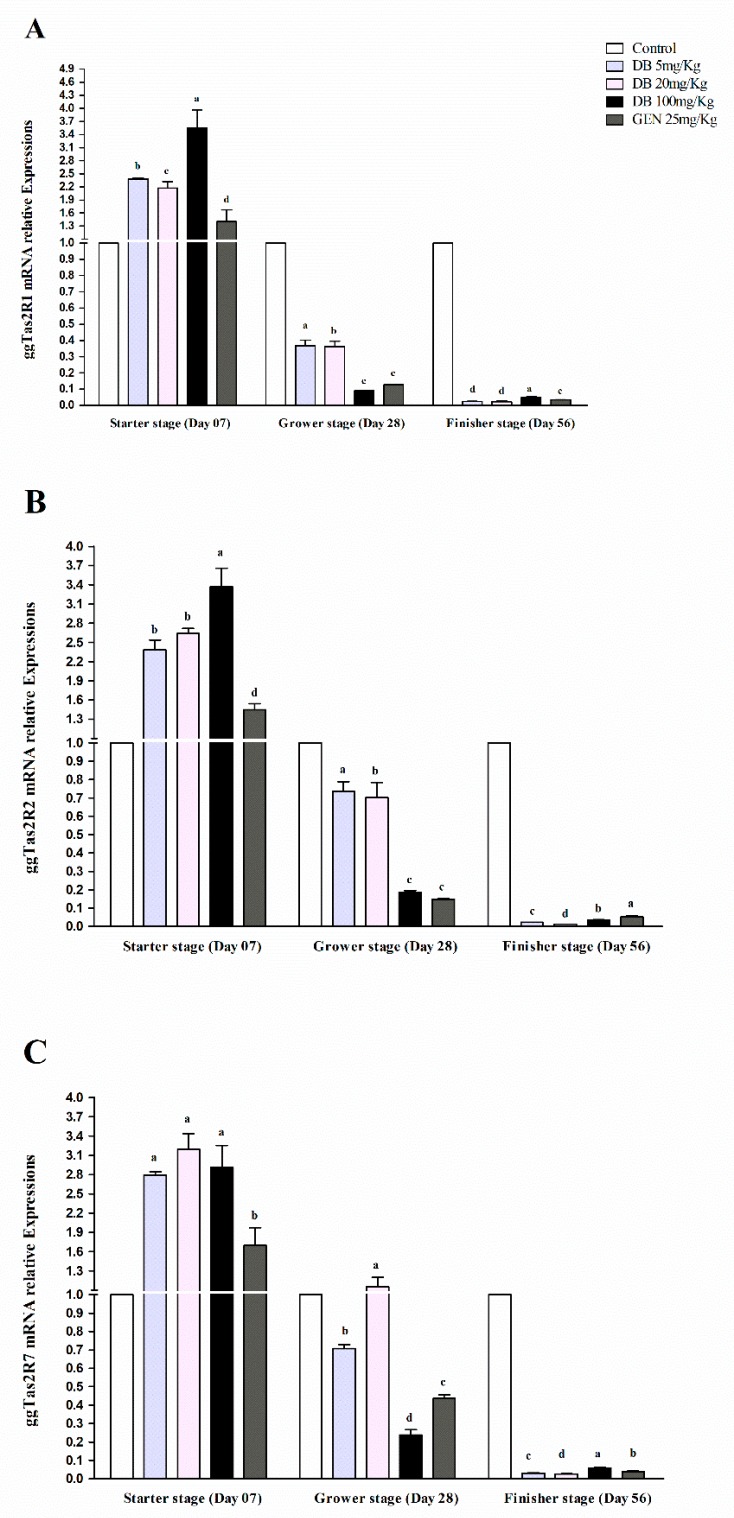
Comparing expressions of bitter taste receptors ((**A**) ggTas2R1, (**B**) ggTas2R2, and (**C**) ggTas2R7), showing their relative mRNA expressions against different doses of DB and GEN in chicken spleens in different growth stages (Day 7, Day 28, and Day 56). The relative mRNA abundance of ggTas2Rs in different growth stages with the spleen serving as the control (relative expression set to 1; n = 6). Values are presented as the mean of relative expressions ± SEM. Differences between groups within a gene mean that those without a common letter differ significantly (*p* < 0.05); differences between the tested tissue (spleen) and the control tissue (spleen) within a gene means that those with marks (a, b, c) differ significantly (*p* < 0.05) from the control group by ANOVA.

The ggTas2Rs expressions in the spleens of chickens were quite highly and significantly (*p* < 0.05) expressed, and the responsiveness expressions were dose-dependent in the starter stage among all the growth stages, respectively (Figure 2A–C). For the comparison of the individual treatments, the ggTas2R1 and ggTas2R2 in DB 100 mg/kg (High Dose) in the starter stage was highly expressed among the other groups and growing stages (Figure 2A,B), whereas ggTas2R7 was highly expressed in the DB Medium-Dose group in the starter stage among all the other treatments in all the stages of growing, respectively (Figure 2C). Expressions of ggTas2Rs were dose-dependent to different DB doses and GEN in the starter stage (Figure 2A–C). However, it was also observed that the ggTas2R7 receptor was highly dose-dependent to the medium dose of DB in the grower stage, and the expressions for all the treatments subsequently decreased in the grower and finisher stages, respectively (Figure 2A–C). Furthermore, ggTas2Rs were gradually less expressed in the DB High-Dose and GEN groups in the grower stage and surprisingly increased in the finisher stage compared to other treatments within the stage. Finally, all the responsiveness expressions were absolutely descendant in the finisher stage, respectively (Figure 2A–C).

#### 3.2.3. mRNA Responsiveness Expressions of ggTas2Rs in Chicken Lungs

**Figure 3:**
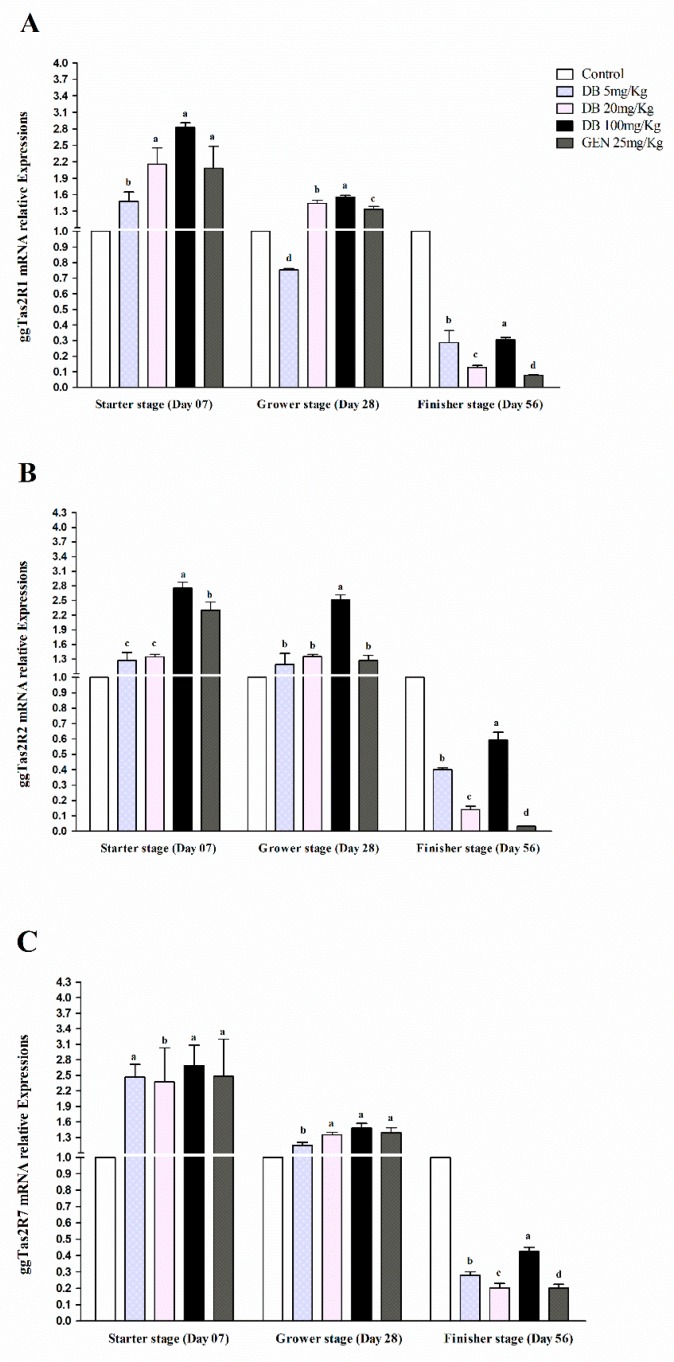
Comparing expressions of bitter taste receptors ((**A**) ggTas2R1, (**B**) ggTas2R2, and (**C**) ggTas2R7), which are showing their relative mRNA expressions against different doses of DB and GEN in chicken lungs in different growth stages (Day 7, Day 28, and Day 56). The relative mRNA abundance of ggTas2Rs in different growth stages with the lung serving as the control (relative expression set to 1; n = 6). Values are presented as mean of relative expressions ± SEM. Differences between groups within a gene mean that those without a common letter differ significantly (*p* < 0.05); differences between the tested tissue (lung of chicken) and the control tissue (lung) within a gene mean that those with marks (a, b, c) differ significantly (*p* < 0.05) from the control group by ANOVA.

The expressions of ggTas2Rs receptors in chicken lungs were significantly (*p* < 0.05) higher in the starter stage than in the grower and finisher stages, respectively (Figure 3A–C). The ggTas2R1 receptor in the DB 100 mg/kg group in the starter stage had potentially higher expression among the groups (Figure 3A). However, ggTas2R2 and ggTas2R7 were significantly (*p* < 0.05) comparably expressed in the grower and finisher stages within the treatments (Figure 3B,C). The responsiveness expressions of ggTas2Rs against DB different doses and GEN in three growing stages of Fast Yellow Chicken was almost equal and dose-dependent with negligible high expressions and variations in the group given DB 100 mg/kg (High-Dose). Therefore, the expressions subsequently decreased in the finisher stage for all the groups, respectively (Figure 3A–C). 

#### 3.2.4. mRNA Responsiveness Expressions of ggTas2Rs in Chicken Kidneys

**Figure 4:**
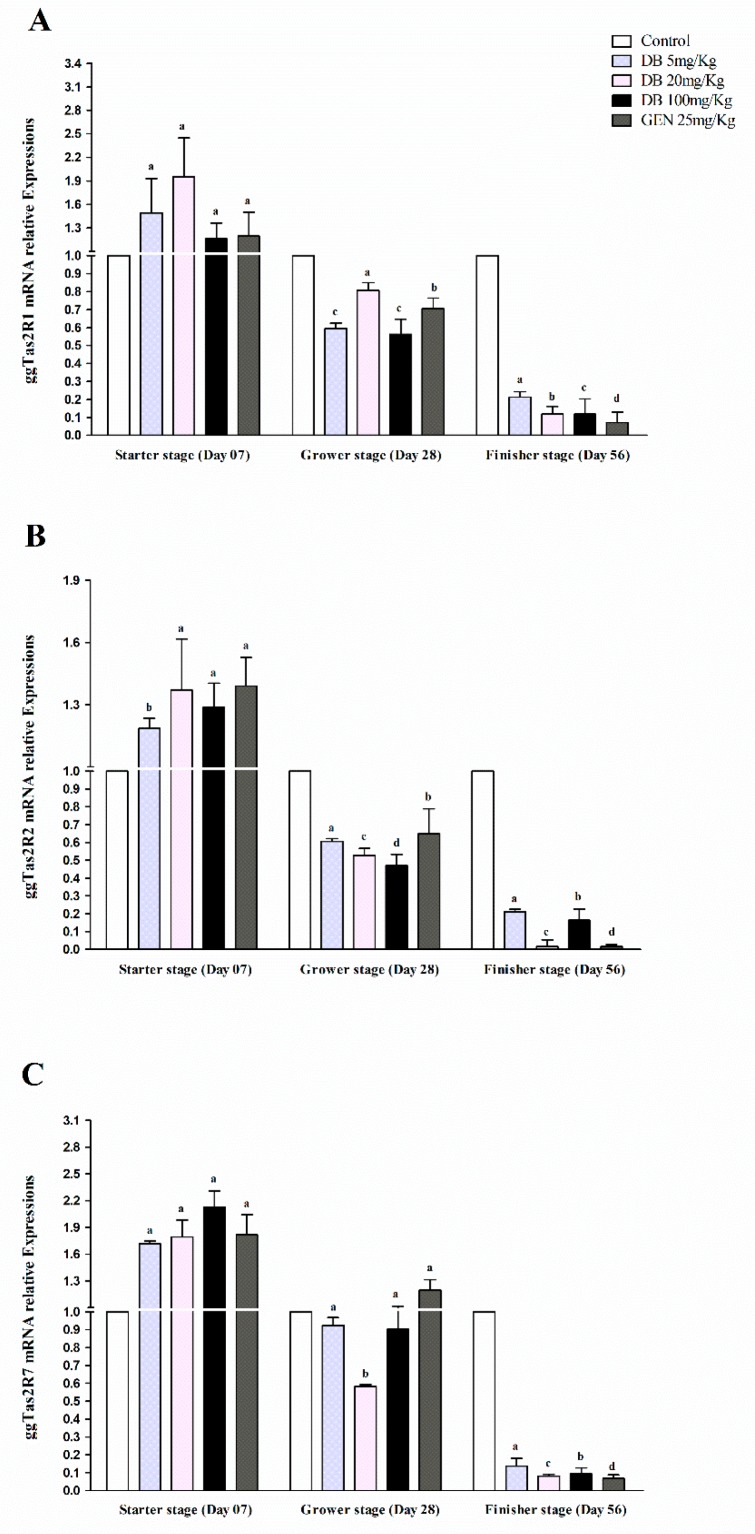
Comparing expressions of bitter taste receptors ((**A**) ggTas2R1, (**B**) ggTas2R2, and (**C**) ggTas2R7) showing their relative mRNA expressions against different doses of DB and GEN in chicken kidneys in different growth stages (Day 7, Day 28, and Day 56). Relative mRNA abundance of ggTas2Rs in different growth stages with the kidney serving as the control (relative expression set to 1; n = 6). Values are presented as the mean of relative expressions ± SEM. The differences between groups within a gene mean that without a common letter differ significantly (*p* < 0.05); differences between the tested tissue (chicken kidney) and the control tissue (kidney) within a gene means that those with marks (a, b, c) differ significantly (*p* < 0.05) from the control group by ANOVA.

The expressions of ggTas2Rs in chicken kidneys showed significantly (*p* < 0.05) high expressions against the GEN and DB Medium-Dose group with the exception of ggTas2R7, which was higher expressed in the DB High-Dose group in the starter stage, and then gradually less expressed than the other ggTas2Rs in the grower and finisher stages, respectively (Figure 4A–C). These results showed that ggTas2R1, ggTas2R2, and ggTas2R7 have contrary expressions levels in kidneys at different chicken growth stages (Figure 4A–C). For instance, it was found that the responsiveness expressions of ggTas2Rs were dose-dependent against DB different doses and GEN in the starter stage, while ggTas2R1 expressions were highly dose-dependent to DB 20 mg/kg in the starter stage and dose-independent in all the groups in the finisher stage (Figure 4A). However, ggTas2R2 response was highly dose-dependent to GEN in the starter stage and dose-independent in the grower and finisher stages for the mentioned gene (Figure 2B). The ggTas2R7 expression was approximately dose-dependent to all the treatments in the starter stage, but only to GEN in the grower stage (Figure 4C). In the finisher stage, neither of the expressions were dose-dependent and showed dose-independent responsiveness against all the treatments, respectively (Figure 4A–C).

#### 3.2.5. mRNA Responsiveness Expressions of ggTas2Rs in Chicken Bursa Fabricius

**Figure 5:**
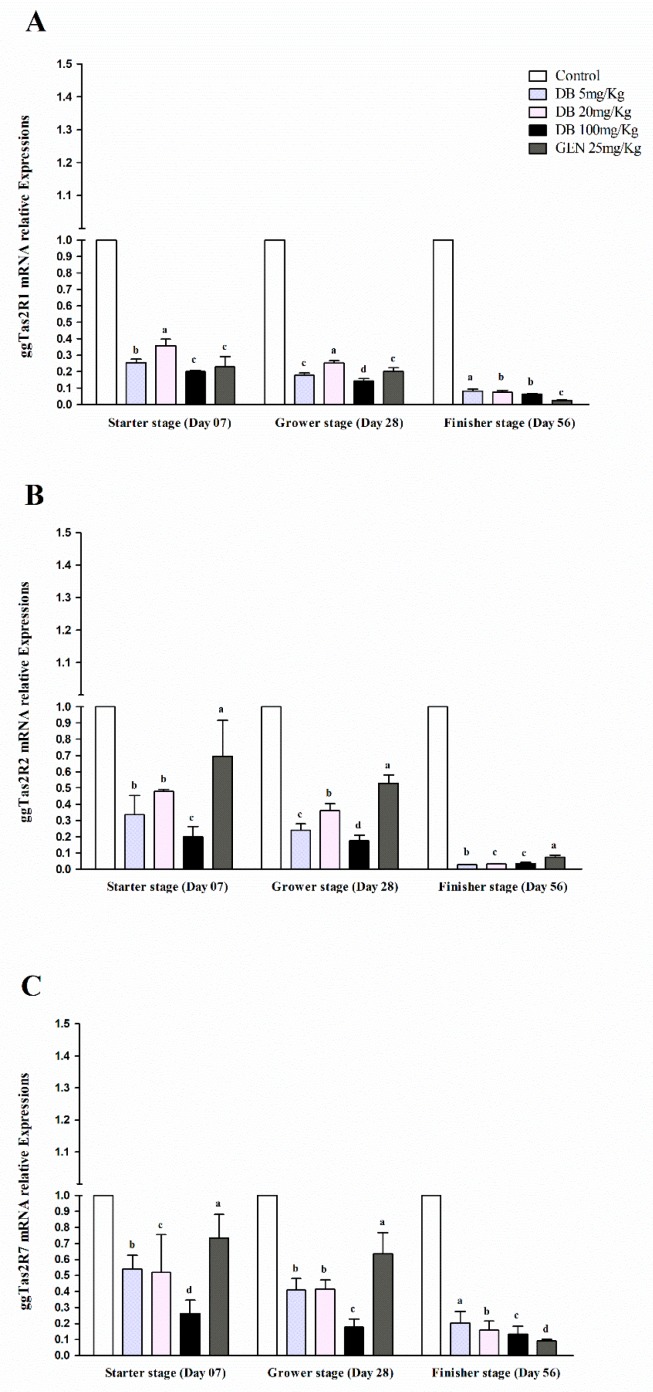
Comparing expressions of bitter taste receptors ((**A**) ggTas2R1, (**B**) ggTas2R2, and (**C**) ggTas2R7), and showing their relative mRNA expressions against different doses of DB and GEN in the bursa Fabricius of chicken at different growth stages (Day 7, Day 28, and Day 56). Relative mRNA abundance of ggTas2Rs in different growth stages with the bursa Fabricius serving as the control (relative expression set to 1; n = 6). Values are presented as mean of relative expressions ± SEM. Differences between groups within a gene mean that those without a common letter differ significantly (*p* < 0.05); differences between the tested tissue (chicken bursa Fabricius) and the control tissue (bursa Fabricius) within a gene mean that those with marks (a, b, c) differ significantly (*p* < 0.05) from the control group by ANOVA.

The expressions of ggTas2Rs in the bursa Fabricius of Fast Yellow Chicken were significantly (*p* < 0.05) lower expressed than the control group in different growth stages (Figure 5A–C). Regarding each gene’s individual expressions level, the mRNA expressions in the GEN group were significantly (*p* < 0.05) highly expressed in all the growing stages except for ggTas2R1, which was highly expressed in DB 20 mg/kg (the Medium-Dose group) in the starter stage. The responsiveness expression against dose was independent in bursa Fabricius and had a relatively low response in the starter and grower stages, but all of the expressions subsequently decreased in the finisher stage (Figure 5A–C). The dose-dependent responsiveness in bursa Fabricius at the starter/grower stages was a result of the subjection to GEN (Figure 5A–C).

## 4. Discussion

Over the past years, tremendous progress has been made investigating the wide-ranging expression of bitter taste receptors (Tas2Rs) inside the vertebrate’s various tissues and their bitter taste perception. Interestingly, numerous tissues in addition to gustatory and non-gustatory tissues have been identified to express taste receptor molecules. These findings bear imperative implications for the roles that taste receptors fulfill in vertebrates, which are currently envisioned much broader than previously thought [44]. The sense of taste facilitates the recognition of beneficial or potentially poisonous and harmful food ingredients prior to ingestion [55]. Bitter taste perception in vertebrates relies on the Tas2R genes, ranging from only three in chicken to over 50 in frogs. Possessing a low repertoire of Tas2Rs makes the chicken an appropriate candidate for a model animal in the study of different aspects regarding bitter taste. Furthermore, the agricultural reputation for finding bitter tastants in chicken feedstuff is countless, since their nutrition may be enhanced due to lack of aversiveness [17,43].

To our knowledge, this is the first research to evaluate the ggTas2Rs expression responses against different doses of denatonium benzoate and genistein in the heart, spleen, lung, kidney, and bursa Fabricius of chickens. Several studies reported that in vertebrates, the sensors for bitter compounds are taste receptors (T2Rs or Tas2Rs); basically, they are distributed in the taste receptor cells of taste buds of an oral cavity belonging to the G-protein-coupled receptors super family (GPCRs). In chickens, taste sensing research has mostly focused on taste bud morphological distribution, development, and various tastants’ thresholds [17,35,43,45,46]. However, in mammals, it is well established that the expressions of Tas2Rs and their downstream signaling molecules and taste-related genes have been found in various extraoral systems such as the respiratory, digestive, and genitourinary systems, as well in brain and immune cells. These receptors are functional in different body locations with varied biological regulatory mechanisms [36]. The extra-gustatory Tas2Rs receptors have been concerned in diverse functions, representing cellular responses to poisons and toxins [56]. This suggests that bitter composition sensing has a physiological role beyond food evaluation and consumption. Furthermore, in chickens, the gustatory and extra-gustatory mechanisms of involving taste signaling have been recently shown [57]. In this study, the expression levels of different DB doses with GEN were varied, and a high dose of DB was comparatively higher expressed among different doses. Therefore, the study demonstrated the responsiveness expressions of ggTas2Rs (ggTas2R1, ggTas2R2, and ggTas2R7) against DB and GEN in five (5) essential organs of the local Chinese Fast Yellow Chicken.

Several studies reported that the expressions of taste-related genes demonstrated their involvement in gustatory and extra-gustatory tissues; furthermore, the expression was also evaluated as a taste transduction gene in chickens [58,59,60]. In the current experiment, these bitter taste-related genes, which were determined by qRT-PCR, were found at different expression levels in all the organs in the starter, grower, and finisher stages with varied responsiveness expressions against different doses of DB and GEN over 56 consecutive days of growing. Furthermore, the expressions were sufficiently higher at the starter and grower stages of growing, and then consequently decreased in the finisher stage. However, the organ weight gained adequately improved for all the treatments as predicted; beyond seven days, the chicken organ weights increased collaboratively with feed consumption, and bitterness sensitivity subsequently decreased. Therefore, the bitterness sensitivity in chickens is dependent on age. It was reported earlier that in chickens, bitterness susceptibility is dependent on the age of the chicken, as bitter taste receptors were highly expressed in zero to one-week-old chicks and dependently decreased in aged chickens, and these behavioral responses were conserved since hatching to the maturing period [19,61]. However, insufficient research has been done to investigate growth-related taste perceptions and their subsequent effect on the animal’s growth [61,62]. Bitter molecules detected by the ggTas2R family of G-protein-coupled receptors (GPCRs) were involved in chickens perceiving potentially toxic compounds [63]. As described earlier, chickens in the initial period of growing are more susceptible against salt, sour, and bitterness than those entering the maturing stage [64,65]. Therefore, we also found that chicks in the starter/grower stages were more sensitive than those in the finisher stage, and the responsiveness expressions of bitter receptors were correlatively high in the starter/grower stages than in the finisher stage of growing. In addition to this, it has been reported that the human bitter taste receptor hTAS2R39 seems to be a bitter receptor agonist for many dietary compounds, such as isoflavones from soy bean [66] and many other flavonoids from several plant sources and synthetic denatonium benzoate [67,68]. The birds fed with 40 to 80 mg/kg of genistein revealed the greater relative weight gains of thymus and bursa Fabricius; however, the spleen weight was not affected. Genistein supplementation not only improved growth performance, it also could beneficially affect immunological responses in broiler chicks [69]. Additionally, studies have shown that genistein improves kidney function and weight gain [70]. Therefore, our study also indicated that genistein improved the organ growth performance and may have a potential influence on the regulation of bursal immunity and kidney function.

In recent years, several reports on the extra-gustatory expression of taste receptors obviously suggested that their role is not only limited to taste perception [17,36,45,71]. Meanwhile, the expression of taste receptor genes and functions have been identified in the gastrointestinal and respiratory tracts of mammals, in the male reproductive system, as well as in the brain and heart [19,72,73,74]. Seemingly, the responsiveness expressions of the ggTas2Rs bitter receptors in the chicken organs were also found with distinguished expression levels in different stages of growth; these contributions have received the most attention from researchers in recent years. Bitter taste receptor expression is not restricted to the upper respiratory tract; it extends into the lower respiratory tract [75]. It should be acknowledged that Tas1r gene expression has been detected in the respiratory system of rodents [76]. qRT-PCR analyses showed that rat neonatal whole heart cDNA, the seven bitter taste receptor genes, as well as two genes encoding the umami receptor subunits, Tas1r1 and Tas1r3, were found to be expressed. Moreover, samples of ventricular tissue of failing hearts were tested and revealed the expression of more than half of all human Tas2Rs genes [13,32]. Remarkably, the mRNA responsiveness expressions of the chicken bitter taste receptor Tas2Rs against DB different doses and GEN, which are known to be extensively represented in the heart, spleen, and lungs of chickens. In comparison, the different DB doses with GEN, the responsiveness expressions against DB 100 mg/kg (High Dose) and GEN were highly dose-dependent in the heart, spleen, lung, kidney, and bursa Fabricius in the starter and grower stages. However, dose-independent lower responsiveness expressions were found in the finisher stage with some exceptions in bursa Fabricius. In summary, the results of the present study indicate the significantly higher expression of bitter taste responsive genes in the starter and grower stages among all the organs except for bursa Fabricius. The Tas2Rs receptors were highly expressed in the heart, spleen, and lung, but lower in the bursa Fabricius in the experimental period among the organs. These findings prove and suggest that bitterness sensitivity decreases as chickens age. However, baby chicks were found to be more sensitive to bitterness than adults. The mentioned findings may be useful in the production of new feedstuffs for chicken depending on their growth stages. Hereafter, further research studies are required to investigate bitter receptors’ varied expressions in body organs, the physiological and functional effects of bitter taste receptors in the non-gustatory organs of the chicken, and the molecular mechanism pathways involved in bitter responsiveness and their role involved in the regulation of bronchodilation, heart functions, kidney functions, and the immunity of bursa Fabricius in chickens.

## 5. Conclusions

Our study demonstrated that the responsiveness expressions of ggTas2Rs (ggTas2R1, ggTas2R2, and ggTas2R7) against denatonium benzoate at different doses were higher in the lungs, spleen, and heart, but lower in bursa Fabricius among the organs. The responsiveness expressions were highly dose-dependent in the starter and grower stages of the heart, spleen, lungs, and kidneys, but dose-independent in the bursa Fabricius in different growing stages of local Chinese Fast Yellow Chicken; bitterness sensitivity decreased subsequently. However, organ weight gains were impaired in the group that received a high dose of denatonium benzoate, and the researchers observed that chickens have a lower tolerance for high-dose denatonium benzoate feed. These findings are valuable for clinicians and pharmacologists because of ggTas2Rs-wide extraoral expressions, as taste biology is directly correlated to diseases, and may affect kidney and heart functions, the regulatory mechanisms of lungs, and the immunity pathways of bursa Fabricius. It may also help with nutritionist and benefit feed industries to improve the production of new feedstuffs for chicken according to their growing stages.

## Figures and Tables

**Table 1:** Feed formulation for the entire period of the experiment (d 1–56).

Item	Diet	
	D 1 to 28	D 28 to 56
Ingredient (%)		
Corn	61.52	74.00
Soybean meal	29.00	12.00
Soybean oil	2.44	2.60
Corn gluten meal	2.00	7.32
Dicalcium phosphate	1.68	1.02
Premix	1.50	1.00
Limestone	1.15	1.05
Lysine sulfate	0.51	0.80
Methionine	0.20	0.21
**Total**	**100**	**100**
Calculation of nutrients		
Metabolizable energy, MJ/kg	11.92	12.13
Crude protein, %	21.00	19.00
Lysine, %	1.10	0.97
Methionine, %	0.46	0.40
Methionine + cystine, %	0.80	0.72
Calcium, %	1.00	0.90
Available phosphorus, %	0.70	0.65

Provided the following % per kilogram in completed diet: vitamin A, 12,500 IU; vitamin D3, 2500 IU; vitamin E, 30 IU; vitamin K3, 2.65 mg; vitamin B_1_, 2 mg; vitamin B_2_, 6 mg; nicotinic acid, 50 mg; pantothenic acid, 12 mg; vitamin B_6_, 4 mg; folic acid, 1.25 mg; vitamin B_12_, 0.025 mg; biotin, 0.25 mg; Fe, 50 mg; Zn, 75 mg; Mn, 100 mg; Cu, 8 mg; I, 0.35 mg; Co, 0.2 mg; and Se, 0.15 mg.

**Table 2:** Primers used for real-time PCR analysis of genes expressions.

Target	Accession No	Forward Primer	Reverse Primer	Product Size (bp)
ggTas2R1	AB249766.1	GGTGCCATCAAGACAGTCTTCTC	ACAGGCAGCCACTACAACAACA	135
ggTas2R2	AB249767.1	GCGATGATTCCATGGCTGC	CGTTGACCTGCAGAGGTAGG	102
ggTas2R7	NM_001080719.1	TGGCAGAGCAGCACAACACAAC	TACAAGACGCAGCCACAATGAA	111
β-actin	NM_205518.1	CCAGCCATGTATGTAGCCATCCAG	ACGGCCAGCCAGATCCAGAC	162

gg–Gallus Gallus; TasR-Taste Receptor, β-actin-Housekeeping gene.

**Table 3:** Live body weight and organ weights in the starter, grower, and finisher stages.

Stages	Treatments	Heart (g)	Spleen (g)	Lungs (g)	Kidneys (g)	Bursa Fabricius (g)	Live Body Weight (g)
Starter	Control	0.64 ± 0.04^b^	0.32 ± 0.05^a^	0.52 ± 0.01^b,c^	0.72 ± 0.17^a^	0.21 ± 0.30^a^	70.94 ± 0.79^b^
	DB-Low Dose	0.66 ± 0.02^b^	0.25 ± 0.03^b^	0.52 ± 0.02^b,c^	0.70 ± 0.10^b^	0.09 ± 0.03^b^	76.34 ± 0.41^a^
	DB-Medium Dose	0.51 ± 0.04^c^	0.22 ± 0.01^c^	0.63 ± 0.04^a^	0.68 ± 0.08^b,c^	0.09 ± 0.04^b^	63.64 ± 0.88^c^
	DB-High Dose	0.43 ± 0.02^d^	0.19 ± 0.03^c,d^	0.47 ± 0.09^d^	0.65 ± 0.08^c^	0.09 ± 0.02^b^	60.02 ± 0.98^d^
	GEN	0.69 ± 0.07^a^	0.34 ± 0.04^a^	0.57 ± 0.01^b^	0.73 ± 0.05^a^	0.09 ± 0.01^b^	63.70 ± 0.99^c^
Grower	Control	2.09 ± 0.28^b,c^	0.58 ± 0.24^a^	1.75 ± 0.17^b^	2.46 ± 0.08^a,b^	0.55 ± 0.02^b^	255.00 ± 1.33^a^
	DB-Low Dose	2.13 ± 0.13^b^	0.56 ± 0.15^b^	1.31 ± 0.13^c^	2.14 ± 0.09^b^	0.57 ± 0.03^a^	220.50 ± 2.14^b^
	DB-Medium Dose	1.71 ± 0.24^c^	0.37 ± 0.13^c^	1.84 ± 0.23^a^	2.13 ± 0.04^b^	0.47 ± 0.02^c^	197.10 ± 3.12^d^
	DB-High Dose	1.63 ± 0.27^d^	0.31 ± 0.14^d^	1.29 ± 0.07^d^	2.11 ± 0.06^c,d^	0.48 ± 0.02^c^	197.40 ± 2.13^d^
	GEN	2.37 ± 0.19^a^	0.45 ± 0.19^b,c^	1.79 ± 0.11^b^	2.56 ± 0.04^a^	0.41 ± 0.02^d^	208.30 ± 1.91^c^
Finisher	Control	6.41 ± 0.02^b^	2.24 ± 0.17^b^	4.75 ± 0.11^b,c^	8.58 ± 0.03^a^	2.26 ± 0.09^b,c^	888.20 ± 2.12^a^
	DB-Low Dose	6.92 ± 0.02^a^	2.36 ± 0.25^a^	3.97 ± 1.13^c^	7.87 ± 0.02^b^	2.60 ± 0.05^a^	687.00 ± 2.13^c^
	DB-Medium Dose	5.29 ± 0.03^c^	1.79 ± 0.19^c^	4.86 ± 1.31^a^	7.77 ± 0.06^c^	2.18 ± 0.08^d,e^	725.30 ± 1.93^b^
	DB-High Dose	5.27 ± 0.08^c,d^	1.59 ± 0.32^d^	3.66 ± 0.16^d^	7.22 ± 0.02^d^	2.21 ± 0.03^c^	686.50 ± 1.75^d^
	GEN	6.68 ± 0.03^a,b^	2.32 ± 0.16^a^	4.77 ± 0.50^a,b^	8.62 ± 0.04^a^	2.20 ± 0.02^c^	620.50 ± 2.12^e^
*P*-Value	Starter	<0.001	<0.001	<0.001	<0.001	0.004	0.0047
	Grower	0.005	0.004	0.005	0.004	0.005	0.0038
	Finisher	0.001	0.005	0.001	0.003	0.004	0.0044
	Feed (Treatments)	<0.001	<0.001	<0.001	<0.001	<0.001	<0.001
	Feed × Stages	0.183	0.181	0.111	0.094	0.917	<0.001

Heart, spleen, lung, kidney, and bursa Fabricius weight unit (g, n = 10); the table shows different treatments, DB 5 mg/kg (Low Dose), DB 20 mg/kg (Medium Dose), DB 100 mg/kg (High Dose), and GEN 25 mg/kg, values shown are mean ± SE. (Standard Errors), ^a–e^ means in a row with different superscript differ significantly (*p* < 0.05). The table also shows the interaction between stages and feed. DB: denatonium benzoate; GEN: genistein.
